# Napsin A levels in epithelial lining fluid as a diagnostic biomarker of primary lung adenocarcinoma

**DOI:** 10.1186/s12890-017-0534-z

**Published:** 2017-12-12

**Authors:** Akifumi Uchida, Takuya Samukawa, Tomohiro Kumamoto, Masahiro Ohshige, Kazuhito Hatanaka, Yoshihiro Nakamura, Keiko Mizuno, Ikkou Higashimoto, Masami Sato, Hiromasa Inoue

**Affiliations:** 1 Department of Pulmonary Medicine, Graduate School of Medical and Dental Sciences, Kagoshima University, 8-35-1 Sakuragaoka, Kagoshima, 890-8520 Japan; 2 Department of Pathology, Tokai University School of Medicine, 143 Shimokasuya, Isehara, Kanagawa Japan; 3 Department of General Thoracic Surgery, Graduate School of Medical and Dental Sciences, Kagoshima University, 8-35-1 Sakuragaoka, Kagoshima, Japan

**Keywords:** Lung cancer diagnosis, Primary lung adenocarcinoma, Epithelial lining fluid, Biomarkers, Bronchoscopy

## Abstract

**Background:**

It is crucial to develop novel diagnostic approaches for determining if peripheral lung nodules are malignant, as such nodules are frequently detected due to the increased use of chest computed tomography scans. To this end, we evaluated levels of napsin A in epithelial lining fluid (ELF), since napsin A has been reported to be an immunohistochemical biomarker for histological diagnosis of primary lung adenocarcinoma.

**Methods:**

In consecutive patients with indeterminate peripheral lung nodules, ELF samples were obtained using a bronchoscopic microsampling (BMS) technique. The levels of napsin A and carcinoembryonic antigen (CEA) in ELF at the nodule site were compared with those at the contralateral site. A final diagnosis of primary lung adenocarcinoma was established by surgical resection.

**Results:**

We performed BMS in 43 consecutive patients. Among patients with primary lung adenocarcinoma, the napsin A levels in ELF at the nodule site were markedly higher than those at the contralateral site, while there were no significant differences in CEA levels. Furthermore, in 18 patients who were undiagnosed by bronchoscopy and finally diagnosed by surgery, the napsin A levels in ELF at the nodule site were identically significantly higher than those at the contralateral site. In patients with non-adenocarcinoma, there were no differences in napsin A levels in ELF. The area under the receiver operator characteristic curve for identifying primary lung adenocarcinoma was 0.840 for napsin A and 0.542 for CEA.

**Conclusion:**

Evaluation of napsin A levels in ELF may be useful for distinguishing primary lung adenocarcinoma.

## Background

Lung cancer is one of the most common malignant tumours and carries a high mortality rate [1]. The recent increased use of chest computed tomography (CT) has resulted in more frequent incidental detection of peripheral lung nodules, and the most common malignant finding is primary lung adenocarcinoma [2, 3]. Some novel tumour markers and imaging tests are useful for lung cancer [4], but these markers have inadequate diagnostic accuracy. Various circulating tumour markers for primary lung adenocarcinoma have been identified. Carcinoembryonic antigen (CEA) and sialyl Lewis Xi antigen (SLX) are clinically established and useful for the management of primary lung adenocarcinoma. However, their values in serum are not suitable for screening or diagnosis of early-stage primary lung adenocarcinoma due to low sensitivity and specificity [5–7].

The pathological diagnosis of primary lung adenocarcinoma is usually based on tissue or cytological samples obtained by bronchoscopy. However, collection of such samples is sometimes difficult due to the location and size of the nodule or the condition of the patient [2, 8]. CT-guided needle biopsy and thoracoscopic lung biopsy are useful alternative methods, although the invasiveness of both procedures is a disadvantage [9, 10]. Therefore, it is crucial to develop novel diagnostic tools, including other biomarkers and techniques for sample collection, for the accurate diagnosis of primary lung adenocarcinoma.

**Fig. 1:**
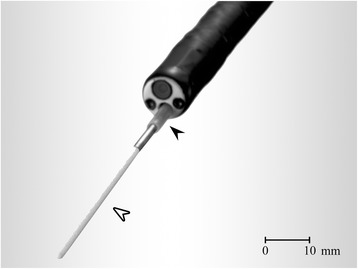
Bronchoscopic microsampling (BMS) procedure. The BMS probe consists of a polyethylene outer sheath (black arrowhead) and an inner cotton fiber rod probe attached to a 30-mm-long stainless steel guide wire (white arrowhead). The probe is guided to the affected lesion in the subsegmental bronchus thorough the bronchoscope channel and the inner fiber rod probe (white arrowhead) is placed on the bronchial membrane as near as possible to the lung nodule. BMS: bronchoscopic maicrosampling

Bronchoscopic microsampling (BMS) has attracted attention as a new diagnostic tool for lung cancer (Fig. 1) [11]. With the BMS technique it is possible to obtain epithelial lining fluid (ELF), the liquid which covers the bronchial wall and alveolus, without the need for saline injection [12]. ELF is transported toward the trachea by ciliary movement, and for diagnostic purposes it is not necessary for ELF to contact the tumour directly. Biochemical substances, including biochemical markers, tumour makers, tumour-derived nucleic acids and drug concentrations, can be measured by analysing ELF without the invasiveness or sample dilution required when testing bronchoalveolar lavage fluid [11, 13–19]. Of note, a previous study reported that measurement of CEA and cytokeratin fragment 19 in ELF was a beneficial diagnostic adjunct in patients with a small peripheral lung nodule [11].

Napsin A, an aspartic protease, is mainly expressed in alveolar type II cells. Previous studies have shown that napsin A is present and active in the alveolar space [20–22]. Immunohistochemical reactivity for napsin A is positive in most cases of primary lung adenocarcinoma, but negative in most cases of squamous cell carcinoma as well as adenocarcinoma of other organs. Its local expression was reported to be useful for identifying the lung origin of metastatic adenocarcinoma [23, 24]. In comparison with napsin A, CEA showed less sensitivity and specificity for histologically diagnosing lung adenocarcinoma using immunohistochemical biomarkers [25].

Based on these findings, we hypothesised that napsin A levels in ELF at the primary lung adenocarcinoma site would be increased. To test this hypothesis, we compared napsin A levels in ELF at the malignant nodule site with those at the unaffected contralateral site. Furthermore, we measured CEA levels in ELF and compared the diagnostic usefulness of ELF-derived napsin A and CEA.

## Methods

### Study design

Patients with an indeterminate peripheral lung nodule who had bronchoscopy at Kagoshima University Medical and Dental Hospital from December 2012 to September 2016 were enroled in this study. We planned to analyse subjects who required surgical resection of the primary lung cancer. The selection criterion for the present study was the presence of a peripheral lung nodule with a diameter of less than 30 mm that was anticipated to be resectable with indications for surgery via bronchoscopy. Written informed consent for this study was obtained before bronchoscopy. This study was approved by the institutional review board of Kagoshima University Medical and Dental Hospital and the committee’s reference number was 24-71. A final definitive histological diagnosis of primary lung adenocarcinoma was made by surgical resection in all patients. For serum marker analysis, healthy volunteers with normal chest radiographs and who had provided written informed consent were matched for gender and age and enrolled in the study as a control group.

### Microsampling probe and procedure

The BMS probe used was the BC-401C (Fig. 1, Olympus Co., Tokyo, Japan). ELF samples were obtained both from a subsegmental bronchus as near as possible to the nodule, using fluoroscopy, and from the contralateral subsegmental bronchus using first the BMS technique followed by endobronchial ultrasound sonography and fluoroscopy with subsequent transbronchial lung biopsy. The BMS method was performed as previously described [12]. After inserting a bronchoscope, the BMS probe in the outer sheath was inserted through the bronchoscope and the inner probe was advanced from the outer sheath toward the affected lesion at the subsegmental bronchus, and then the inner probe was placed on the bronchial membrane for 10 s to absorb the ELF. The inner probe was then immediately withdrawn into the outer sheath. This procedure was repeated three times. We also obtained ELF from the corresponding subsegmental bronchus of the contralateral lung as an internal control. After the BMS procedures, the inner probe tips were frozen at −80 °C. They were weighed and then ELF was extracted by stirring for 1 min following the addition of 3 mL of saline. The tips were then dried and weighed again to measure the ELF volume and calculate the dilution factor.

### Measurement

The napsin A levels in both ELF (ELF-napsin A) and serum (serum-napsin A) were measured by enzyme-linked immunosorbent assay with the Human Napsin A Assay Kit (Immuno-Biological Laboratories Co., Gunma, Japan). The CEA levels in ELF (ELF-CEA) were determined by chemiluminescent enzyme immune assay with Lumipulse Presto CEA (Fujirebio Inc., Saitama, Japan), and those in serum (serum-CEA) were quantified by electro-chemiluminescence immune assay with Cobas 800 (Roche Diagnostics K.K., Tokyo, Japan). Both ELF-napsin A and ELF-CEA were expressed per unit volume after correction for the dilution factor.

### Statistical analysis

The patients who were diagnosed as other than primary lung adenocarcinoma were treated as non-adenocarcinoma. Data were analysed using SPSS version 23 software (IBM SPSS, Chicago, USA). Differences in serum-napsin A and serum-CEA between patients with primary lung adenocarcinoma and those in the control group were analysed by the Mann-Whitney U test. Differences in the variables related to ELF-napsin A and ELF-CEA between the nodule site and contralateral site in the patients who underwent bronchoscopy were compared by the nonparametric Wilcoxon signed-rank test, since the data were not normally distributed. Differences in ELF-napsin A at the nodule site between the patients with primary lung adenocarcinoma and those with non-adenocarcinoma were analysed by the Mann-Whitney U test. The data for each histological subtype of primary lung adenocarcinoma were compared using the nonparametric Kruskal-Wallis analysis of variance test. Correlations were examined using Spearman’s correlation test. Statistical significance was defined as *P* < 0.05. Receiver operating characteristic (ROC) curve analysis was used to assess the ability of ELF-napsin A and ELF-CEA, respectively, to predict the diagnosis of primary lung adenocarcinoma. In ROC analyses, the ELF values at the contralateral site in patients with primary lung adenocarcinoma and at the bilateral sites in patients with non-adenocarcinoma were treated as negative controls. The difference between the areas under the ROC curves (AUCs) for ELF-napsin A and ELF-CEA were performed with EZR version 1.36 (Saitama Medical Center, Jichi Medical University, Saitama, Japan), which is a graphical user interface for R (The R Foundation for Statistical Computing, Vienna, Austria). Any samples with values below the lower limit of quantification were assigned a value of half of the lower quantification limit for analysis.

## Result

### Patients characteristics

We performed BMS in 43 consecutive patients according to our selection criteria. Of 43 patients, 3 patients could not have a surgery because of distant metastasis. Five patients refused surgery for definitive diagnosis. Finally, total 8 patients were excluded, the remaining 35 patients were included in the analysis.

All patients underwent BMS followed by surgical resection without complications, except for mycobacterium infection, allowing for the determination of histological features. Of 35 patients, 27 patients were diagnosed with primary lung adenocarcinoma, and two patients were diagnosed as primary lung adenosquamous carcinoma and combined large cell neuroendocrine carcinoma (LCNEC); LCNEC with adenocarcinoma, respectively. In patients with non-adenocarcinoma, two patients were diagnosed with primary lung squamous cell carcinoma, one patient was diagnosed as primary lung large cell carcinoma, and one patient was diagnosed with metastatic carcinoma from nasopharyngeal carcinoma. The other two patients were identified as mycobacterium tuberculosis and nontuberculous mycobacterial infection, respectively (Table 1).

**Table 1:** Clinical characteristics of patient cohort

Characteristics						
	Primary lung adenocarcinoma	*n* = 27		Non-adenocarcinoma	*n* = 6	
	LC with adenocarcinoma component	*n *= 2				
Age (median, IQR)		67,	12		75,	14
number of patients			(%)	number of patients		(%)
Gender						
Male		15	51.7		4	66.7
Female		14	48.3		2	33.3
Smoking status						
never		13	44.8		2	33.3
ex-smoker		11	38.0		3	50.0
current		5	17.2		1	16.7
Pathological diagnosis						
by bronchoscopy						
yes		11	37.9		5	83.3
no		18	62.1		1	16.7
Pathological stage						
IA		25	86.2			
IB		3	10.3			
IIIA		1	3.5			
Histological subtype and diagnosis						
	Primary lung adenocarcinoma			Primary lung squamous cell carcionoma	2	33.2
	Adenocarcinoma in situ	1	3.5	Primary lung large cell carcionoma	1	16.7
	Minimally invasive adenocaricinoma			Metastatic carcinoma from nasopharyngeal carcinoma	1	16.7
	Non-mucinous	4	13.7			
	Mucinous	1	3.5	Mycobacterium tuberculosis	1	16.7
	Adenocarcinoma			Nontuberculous mycobacterial infection	1	16.7
	Lepidic adenocarcinoma	2	6.8			
	Acinar adenocarcinoma	3	10.3			
	Papillary adenocarcinoma	14	48.2			
	Solid adenocarcinoma	1	3.5			
	Invasive mucinous adenocarcinoma	1	3.5			
	Adenosquamous carcinoma	1	3.5			
	Combined LCNEC	1	3.5			

*LC* Lung carcinoma, *IQR* Interquartile range, *LCNEC* Large cell neuroendocrine carcinoma

Data are presented as number or %, unless otherwise stated

Pathological stage is classified according to the TNM Classification of Malignancy Tumors, 7th Edition

Histological type is categorised according to the WHO Classification of Tumors of the Lung, Pleura, Thymus and Heart, Fourth Edition

**Fig. 2:**
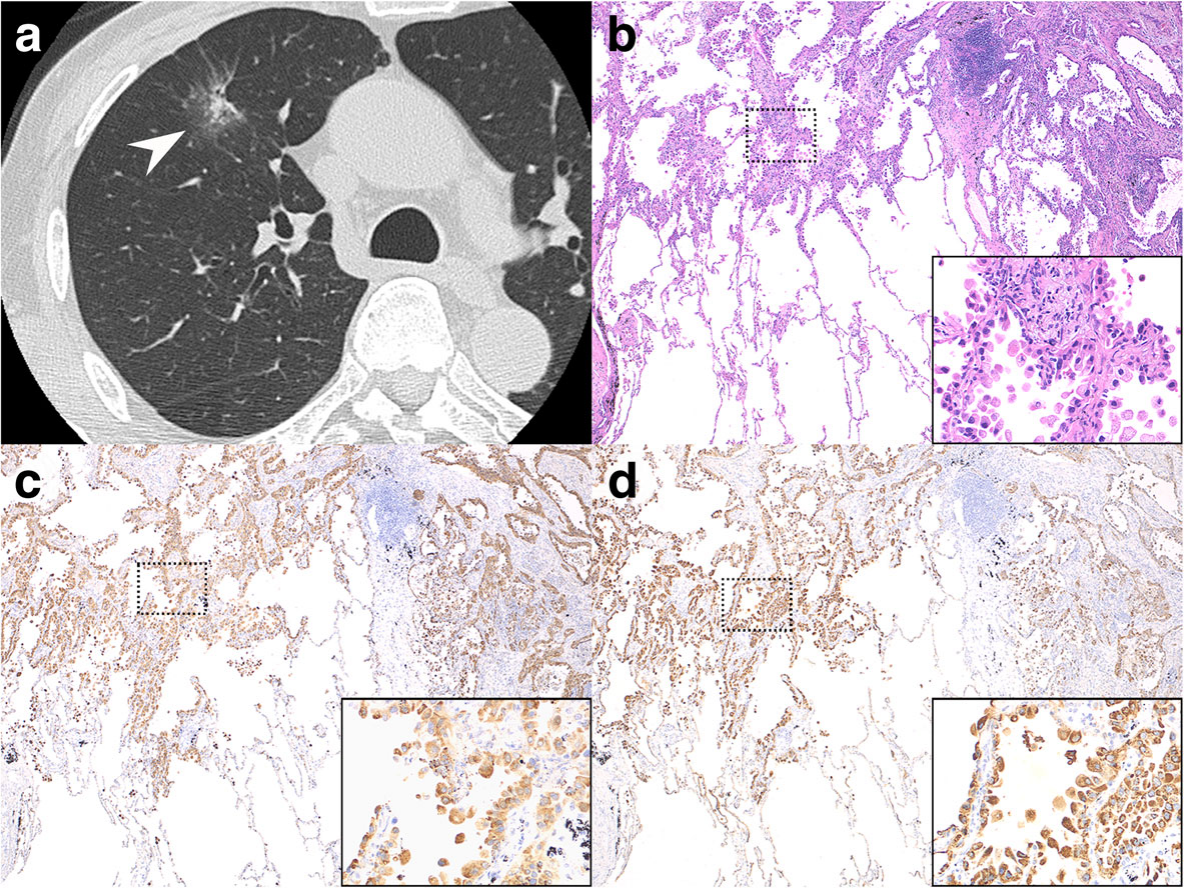
Computed tomography (CT) and immunohistological findings in a representative case. **a** A lung CT image shows a 20-mm, partly solid, ground-glass opacity nodule (arrowhead) in the right upper lobe. **b** Minimally invasive adenocarcinoma. The cancer demonstrates predominant lepidic growth with <5-mm invasion. The cancer cells have polygonal cytoplasm and atypical nuclei (inset) (hematoxylin-eosin staining, original magnification ×40 and high magnification ×100). **c** Immunohistological staining for napsin A. Napsin A is strongly positive in adenocarcinoma cells (inset for greater detail) (original magnification ×40 and high magnification ×100). **d** Immunohistological staining for CEA. CEA is strongly positive in adenocarcinoma cells (inset for greater detail) (original magnification ×40 and high magnification ×100). CT: computed tomography; CEA: carcinoembryonic antigen

Patients with primary adenocarcinoma were 48 to 80 years of age, with a median age of 67 years. In 11 patients, the diagnosis of primary lung adenocarcinoma was obtained by transbronchial lung biopsy or curettage, while the other 18 patients were diagnosed by surgical resection. The pathological stage was determined surgically to be stage IA in 25 patients, stage IB in three, and stage IIIA in one. The diameter of the primary nodule at surgery was 22 (9–36) mm, median (range). One representative case is shown in Fig. 2. This partly solid nodule with a diameter of 20 mm was seen in the right upper lobe on CT. It was not diagnosed by bronchoscopy. ELF-napsin A at the nodule site was 20911 ng∙mL^−1^, and that at the contralateral site was 370 ng∙mL^−1^. In this case, a final diagnosis of primary lung adenocarcinoma was established by surgical resection and napsin A and CEA were strongly expressed in adenocarcinoma cells on immunohistological examination.

### Biological marker levels in ELF and serum

**Fig. 3:**
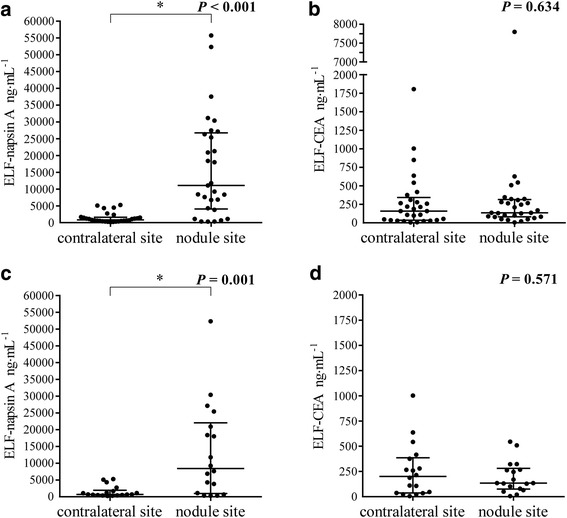
Values of napsin A and CEA in ELF in the patients with primary lung adenocarcinoma. **a** Levels of napsin A in ELF (ELF-napsin A) in the patients with primary lung adenocarcinoma (*n* = 29, *P* < 0.001). **b** Values of CEA in ELF (ELF-CEA) in the patients with primary lung adenocarcinoma (*n* = 29, *P* = 0.634). **c** ELF-napsin A in the patients with primary lung adenocarcinoma who were undiagnosed by bronchoscopy and finally diagnosed by surgery (*n* = 18, *P* = 0.001). **d** ELF-CEA in the patients with primary lung adenocarcinoma who were undiagnosed by bronchoscopy and finally diagnosed by surgery (*n* = 18, *P* = 0.571). CEA: carcinoembryonic antigen; ELF: epithelial lining fluid

Among the patients with primary lung adenocarcinoma, ELF-napsin A at the nodule site was 11091 (326–55729) ng∙mL^−1^ after correction for the dilution factor, while that at the contralateral site was 805 (180–5266) ng∙mL^−1^, indicating a significant difference (*P* < 0.001, Fig. 3a). Conversely, ELF-CEA at the nodule site was 135 (6–7799) ng∙mL^−1^, and that at the contralateral site was 158 (6–1806) ng∙mL^−1^, indicating no significant difference (*P* = 0.634, Fig. 3b). Furthermore, in 18 patients who were undiagnosed by bronchoscopy and finally diagnosed by surgery, ELF-napsin A at the nodule site was 8438 (326–52337) ng∙mL^−1^, and that at the contralateral site was 704 (235–5266) ng∙mL^−1^. In this population there were identically significant differences between ELF-napsin A at the nodule site and those at the contralateral site (*P* = 0.001, Fig. 3c), as well as no difference between ELF-CEA at the nodule site, 135 (6–545) ng∙mL^−1^, and that at the contralateral site, 201 (6–1003) ng∙mL^−1^ (*P* = 0.571, Fig. 3d).

In serum analysis, serum-napsin A in patients with primary lung adenocarcinoma was 16.1 (2.0–366.1) ng∙mL^−1^, and that in the control group was 23.3 (3.1–219.5) ng∙mL^−1^, indicating no significant difference (*P* = 0.251). Similarly, there was no difference between serum-CEA in patients with primary lung adenocarcinoma, 3.3 (0.4-15.7) ng∙mL^−1^, and that in the control group, 1.8 (0.6-9.1) ng∙mL^−1^, (*P* = 0.056).

**Fig. 4:**
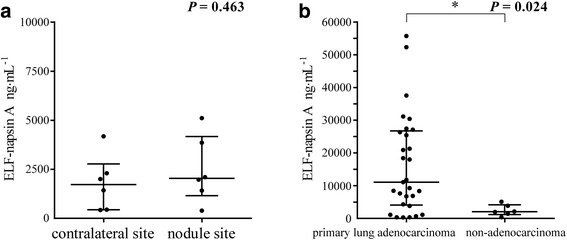
Napsin A levels in ELF in patients with primary lung adenocarcinoma and those with non-adenocarcinoma. **a** Values of napsin A in ELF (ELF-napsin A) in the patients with non-adenocarcinoma (*n* = 6, *P* = 0.463). **b** ELF-napsin A at the nodule site in primary lung adenocarcinoma (*n* = 29) and in non-adenocarcinoma (*n* = 6) (*P* = 0.024). ELF: epithelial lining fluid

In patients with non-adenocarcinoma, ELF-napsin A at the nodule site was 2041 (398–5108) ng∙mL^−1^, and that at the contralateral site was 1719 (425–4186) ng∙mL^−1^, indicating no significant difference (*P* = 0.463, Fig. 4a). ELF-napsin A at the nodule site was significantly higher in primary lung adenocarcinoma than in non-adenocarcinoma (*P* = 0.024, Fig. 4b).

There was no significant difference in ELF-napsin A or ELF-CEA between histological subtypes of primary lung adenocarcinoma (*P* = 0.919 and *P* = 0.590, respectively). There was no significant difference in ELF-napsin A or ELF-CEA between smoking history and pathological stage (data not shown).

### Relationship between ELF and other clinical parameters

**Fig. 5:**
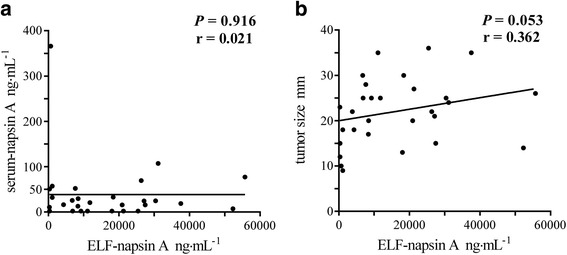
Correlations of levels of napsin A in ELF. **a** Correlation between the levels of napsin A in ELF (ELF-napsin A) and the serum levels of napsin A (serum-napsin A) (*n* = 28, *P* = 0.916, *r* = 0.021). **b** Correlation between ELF-napsin A and the tumour size at surgery (*n* = 29, *P* = 0.053, *r* = 0.362). ELF: epithelial lining fluid

There was no significant correlation between ELF-napsin A and serum-napsin A (*P* = 0.916, *r* = 0.021, Fig. 5a). There was no significant correlation between ELF napsin A and tumour sizes at surgery, however ELF-napsin A tended to rise as tumour sizes increased (*P* = 0.053, *r* = 0.362, Fig. 5b).

### Diagnostic values of napsin A levels in ELF

**Fig. 6:**
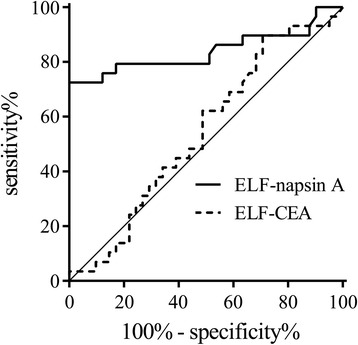
Receiver operating characteristic (ROC) curve for napsin A and CEA in ELF. The ROC curves for napsin A in ELF (ELF-napsin A, solid line) and CEA in ELF (ELF-CEA, dashed line). The areas under the ROC curves are 0.840 for ELF-napsin A and 0.542 for ELF-CEA

We used ROC curve analysis to evaluate the sensitivity and specificity of ELF-napsin A and ELF-CEA as biomarkers for distinguishing primary lung adenocarcinoma (resected from 29 nodule sites) from normal tissue and non-adenocarcinoma (resected from 29 contralateral sites in primary lung adenocarcinoma and 12 bilateral sites in non-adenocarcinoma) (Fig. 6). The AUCs for nodule sites versus negative controls were 0.840 for ELF-napsin A and 0.542 for ELF-CEA (Table 2). The AUC of ELF-napsin A was significantly higher than that of ELF-CEA (*P* < 0.001). It was not possible to perform an ROC analysis for ELF-napsin A in combination with ELF-CEA using a binary logistic regression model, because the *p*-value of an ROC curve analysis for ELF-CEA was not significant. The optimal cut-off values for predicting the diagnosis of primary lung adenocarcinoma were 3280 ng∙mL^−1^ for ELF-napsin A, with a sensitivity and specificity of 79.3% and 82.9%, respectively, and 82 ng∙mL^−1^ for ELF-CEA, with sensitivity and specificity values of 75.9% and 34.1%, respectively (Table 2).

**Table 2:** ROC analysis comparing ELF-napsin A and ELF-CEA

	AUC	95% CI, %	Cut off point	Sensitivity, %	Specificity, %	PPV, %	NPV, %	Positive LR	Negative LR
ELF-napsin A	0.840	72.8 to 95.3	3280 ng∙mL^−1^	79.3	82.9	76.7	85.0	4.64	0.25
ELF-CEA	0.542	40.7 to 67.8	82 ng∙mL^−1^	75.9	34.1	44.9	66.7	1.15	0.71

*ELF* Epithelial lining fluid, *CEA* Carcinoembryonic antigen, *AUC* Area under the ROC curve, *CI* Confidence interval, *PPV* Positive predictive value, *NPV* Negative predictive value, *LR* Likehood ratio

## Discussion

In this study, we demonstrated that ELF-napsin A was a useful diagnostic marker for primary lung adenocarcinoma. ELF-napsin A at the nodule site was significantly higher in patients with primary lung adenocarcinoma. Moreover, except for three patients, ELF-napsin A at the nodule site was higher than that at the contralateral site. In the representative case shown in Fig. 2, many partly solid nodules could not be recognized on fluoroscopy or ultrasound sonography and were undiagnosed by bronchoscopy. Even in such patients, however, ELF-napsin A was significantly elevated. These findings suggested that in patients with elevated ELF-napsin A and no histological diagnosis of primary lung carcinoma by bronchoscopy, it is advisable to perform further evaluation with a high suspicion of primary lung adenocarcinoma. Furthermore, in ROC curve analysis, the AUC for ELF-napsin A was clearly higher than that for ELF-CEA, proving that ELF-napsin A is more valuable for diagnosing primary lung adenocarcinoma.

Because of the small diameters and complicated divergence patterns of bronchi, the detection rate of small lung nodules by bronchoscopy is not high. While the use of fluoroscopy, ultrasound sonography, and new bronchoscopy devices has gradually increased the rate of successful bronchoscopic diagnosis [8, 26–28], it is still not sufficient. If a definite diagnosis is not obtained by bronchoscopy, CT-guided needle biopsy or thoracoscopic lung biopsy are necessary, but both procedures are highly invasive and carry risks of grave complications [9, 10]. It is desirable to develop a diagnostic tool with fewer complications, more convenience, and higher sensitivity and specificity.

The BMS technique, used to obtain ELF with bronchoscopy, is less invasive than bronchoalveolar lavage or transbronchial biopsy. Tumour-derived proteins and nucleic acids are contained in ELF close to a nodule, and the ELF is then transported toward the central bronchi by ciliary movement. As a result, the characteristics of a nodule can be evaluated by investigating ELF and direct access to the nodule is not necessary [11, 17–19]. A previous report found that the measurement of three biomarkers in ELF, namely CEA, cytokeratin fragment 19 and SLX, were useful for diagnosis of a small lung nodule [11]. But since these tumour markers are not specific for primary lung carcinoma, their utility for distinguishing primary lung carcinoma from metastatic cancer is uncertain. In contrast, napsin A is used as a diagnostic biomarker in immunohistochemical analyses for the purpose of discriminating between primary and metastatic lung carcinoma [23, 24].

In the present study, we demonstrated that ELF-napsin A at nodule sites was significantly higher than that at unaffected contralateral sites. The mechanism underlying this elevation in patients with primary lung adenocarcinoma is not known for certain, but it is probably due to overexpression of napsin A on primary lung adenocarcinoma tissue [29] in the alveolar space [20–22]. It may be that napsin A expressed by primary lung adenocarcinoma is released into the alveolar space and transported toward the central bronchi. The molecular weight of napsin A is approximately 38 kDa [30], while that of CEA is estimated to be 180 kDa [31]. This difference may account for the discrepancy in the detected levels of these markers in ELF. Furthermore, ELF-napsin A at nodule sites tended to rise according to increase in tumour size at surgery, suggesting that the expression of napsin A was greater with increased mass and secretory ability of primary lung adenocarcinoma, regardless of its histological subtype.

Serum-napsin A in patients with primary lung adenocarcinoma was not elevated compared with that in the control group. These results were consistent with our previous findings [32]. CEA is a commonly recommended biomarker in the management of lung cancer [33, 34]. But, because of its low sensitivity, it has not been generally recommended as a tool for the early screening of lung carcinoma, and it has been used as a potential prognostic biomarker and not a diagnostic biomarker. Moreover, CEA is not in itself a sufficiently strong indicator to guide treatment decisions for lung cancer [35]. A combination of biomarkers identifying primary lung adenocarcinoma may be helpful in distinguishing early-stage lung adenocarcinoma from benign lung disease, which presents as suspicious lung nodules.

The current study had several limitations. First, our sample size was small. Second, we could determine ELF-napsin A and ELF-CEA in cases of non-adenocarcinoma, including benign tumours and inflammation, in only a few patients. Last, the median diameter of primary lung adenocarcinoma at surgery was 22 mm. Therefore, our results should be confirmed in a larger cohort study, which includes various diseases and smaller-size lung nodules.

## Conclusion

In summary, elevated napsin A levels in ELF were found in patients with primary lung adenocarcinoma, and the levels tended to rise as tumour size increased at surgery. Our findings suggest that napsin A levels in ELF may be clinically useful for distinguishing primary lung adenocarcinoma.

## Abbreviations

AUC: Areas under the ROC curve
BMS: Bronchoscopic microsampling
CEA: Carcinoembryonic antigen
CT: Computed tomography
ELF: Epithelial lining fluid
ELF-CEA: CEA levels in ELF
ELF-napsin A: Napsin A levels in ELF
ROC: Receiver operating characteristic
serum-CEA: CEA levels in serum
serum-napsin A: Napsin A levels in serum
SLX: Sialyl Lewis Xi antigen
